# Cross‐Sectional and Longitudinal Associations Between Dietary Intake and Depressive Symptoms in Young South African Adults: The African‐PREDICT Study

**DOI:** 10.1155/da/2938819

**Published:** 2026-09-29

**Authors:** Esmé Jansen van Vuren, Adrienne O’Neil, Deborah N. Ashtree, Melissa M. Lane, Rebecca Orr, Marlien Pieters, Tertia van Zyl

**Affiliations:** ^1^ Hypertension in Africa Research Team (HART) North-West University Potchefstroom South Africa nwu.ac.za; ^2^ South African Medical Research Council: Extramural Unit for Hypertension and Cardiovascular Disease North-West University Potchefstroom South Africa nwu.ac.za; ^3^ IMPACT (The Institute for Mental and Physical Health and Clinical Translation) Food and Mood Centre Deakin University Geelong Australia deakin.edu.au; ^4^ Centre of Excellence for Nutrition North-West University Potchefstroom South Africa nwu.ac.za

**Keywords:** calcium, depression, global burden of disease study, nutrition, sugar-sweetened beverages, vegetables

## Abstract

**Introduction:**

Depression is highly prevalent among young adults worldwide. While research links health behaviours, such as dietary intake, to depression, few studies have examined these associations in low‐ and middle‐income countries (LMICs), including South Africa. This study investigated whether dietary intake was associated with risk of depression in a cohort of young South African adults, aged 20–30 years, as part of the Global burden of disease Lifestyle And mental Disorder (GLAD) project.

**Methods:**

This 5‐year prospective cohort study was conducted in the North West Province of South Africa following the GLAD project protocol (DERR1‐10.2196/65576). Dietary exposures were evaluated using three non‐consecutive 24‐h dietary recalls, measuring daily intake of food groups and nutrients defined by the GBD study. Depression outcomes were assessed at baseline (*n* = 1039) and follow‐up (*n* = 551) using the Patient Health Questionnaire (cut‐off ≥10). Logistic and Poisson regression analyses were performed, reporting odds ratios (ORs) and relative risks (RRs). Four models were tested: unadjusted, sociodemographic‐adjusted, total energy (TE) intake‐adjusted and fully adjusted (both sociodemographic and TE‐adjusted). For longitudinal analyses, baseline depression cases were additionally excluded (*n* = 403).

**Results:**

Participants (mean age 24.55 years; 51.4% female; 48.6% Black) had a 29.45% baseline depression prevalence. Higher milk intake was associated with a lower risk of incident depression (RR = 0.94, 95% CI 0.91–0.98) in the TE‐adjusted model. Cross‐sectionally, higher sugar‐sweetened beverage consumption was associated with higher odds of depression, whereas higher calcium (OR = 0.48, 95% CI: 0.31, 0.76) and vegetable intake (OR = 0.74, 95% CI: 0.61, 0.91) were associated with lower odds after TE adjustment. Higher fibre intake was associated with lower odds in the unadjusted model.

**Conclusion:**

Higher milk intake was associated with lower incident depression, while higher calcium, vegetable and fibre intake were associated with lower depression prevalence, but not when adjusting for sociodemographic information. These findings suggest that sociodemographic context should be considered alongside dietary strategies to support prevention efforts for common mental disorders in young adults.

**Trial Registration:** ClinicalTrials.gov identifier: NCT03292094

## 1. Introduction

The global burden of mental disorders is substantial. In 2021, common mental disorders, namely, depressive and anxiety disorders, were ranked among the leading causes of disability‐adjusted life years (DALYs) and were major contributors to years lived with disability [1]. Major depressive disorder (MDD) remains a significant contributor to this burden, accounting for the highest proportional age‐standardised incidence rate (~76%) of all mental disorder subtypes in 2021 [1]. Mental disorder‐related DALYs peak between the ages of 25 and 34 years, highlighting the heightened vulnerability of young adults [2]. Life course data further demonstrate that the onset of depression and anxiety often occurs at younger ages and that age‐specific interventions targeting modifiable risk factors are needed to address the global mental health burden [3].

Given the substantial burden of common mental disorders, dietary intake is a relevant modifiable exposure and is already incorporated within the global burden of disease (GBD) study as a risk factor for non‐communicable diseases, including cardiovascular disease (CVD), which commonly co‐occurs with depression and anxiety [4]. Emerging evidence also suggests that diet may serve as a modifiable risk factor for common mental disorders, such as depression and anxiety [5, 6]. However, lifestyle exposures are not yet routinely reported as risk‐outcome pairs for these common mental health conditions within the GBD framework, limiting evidence‐informed prevention priorities and guideline development, particularly in low‐ and middle‐income countries (LMICs). This gap provides the rationale for the GBD Lifestyle And mental Disorder (GLAD) Taskforce, an international collaborative initiative aiming to integrate lifestyle exposures as risk factors for common mental disorders in the GBD study [7].

A key objective of the GLAD project is to produce evidence on the diet‐common mental disorder association in regions of the world where data are particularly scarce, including LMICs, such as South Africa. This evidence gap is concerning given the rising prevalence of depression in LMICs [2, 8]. In 2021, Sub‐Saharan Africa had one of the highest age‐standardised incidence rates for mental disorders, with Southern Sub‐Saharan Africa reporting the fourth highest proportion of DALYs due to MDD [1]. These trends, alongside a significant dietary transition in Black South Africans from prudent dietary patterns, high in minimally processed plant‐based foods and moderate animal‐based foods, toward more westernised diets high in refined sugars, saturated fats and ultra‐processed food (UPF) [9], underscore the urgent need for context‐specific research. Accordingly, this study aims to investigate whether cross‐sectional and longitudinal associations exist between depression and the consumption of specific food groups and nutrients in a cohort of young South African adults.

## 2. Methods and Materials

### 2.1. Study Design

This study formed part of the GLAD Taskforce [7] and used data from the African Prospective study on the early detection and identification of CVD and hypertension (African‐PREDICT). The African‐PREDICT study was initiated to monitor the development of hypertension in a bi‐racial sample residing in the JB Marks local municipality, North West Province, South Africa, over a 5‐year follow‐up period [10]. The study’s baseline phase was conducted from 2013 to 2017 and included 1202 young individuals aged 20–30 years. Exclusion criteria included clinic brachial blood pressure >140/90 mmHg; HIV infection; previous diagnosis of any of the following chronic diseases: cancer, tuberculosis, liver disease, renal disease, diabetes or CVD; medication use for hypertension, diabetes or HIV; presence of fever; being pregnant or breastfeeding; phobia of needles; inability to read or understand English; and any race other than self‐reported black or white racial groups. The study’s first follow‐up phase commenced in 2018 and was completed in 2024, during which each participant completed a single follow‐up assessment.

For the present analyses, individuals with missing dietary (*n* = 31) or depressive symptom (*n* = 132) data at baseline were excluded, resulting in a baseline analytical sample of 1039 individuals. Of these individuals, 710 were successfully followed up, after which individuals with missing depressive symptom data at follow‐up (*n* = 159), resulting in a final longitudinal sample of 551 individuals, as shown in Figure 1. For incident depression analyses, those who met depression criteria at baseline (refer to description later in this study) were further excluded (*n* = 148).

**Figure 1:**
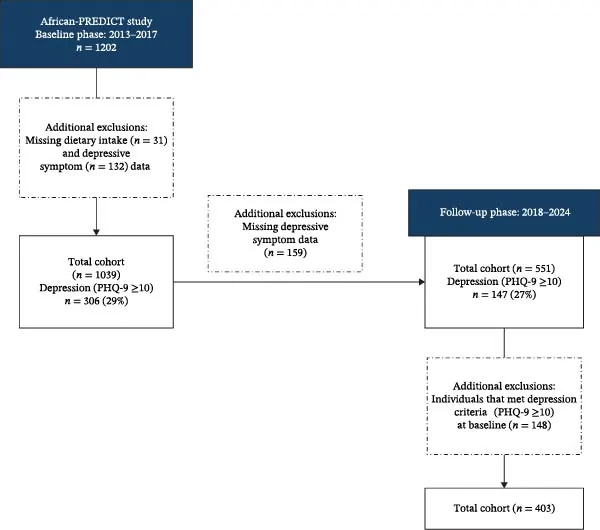
Study design flow diagram.

The African‐PREDICT study complies with the Declaration of Helsinki, was approved by the Health Research Ethics Committee of the North‐West University (NWU‐00001‐12‐A1). Written informed consent was provided by all participants prior to data collection.

### 2.2. Organisational Procedures

Screening procedures were conducted to determine eligibility for participation according to the aforementioned inclusion and exclusion criteria at baseline. Eligible participants were invited to the Hypertension Research and Training Clinic, situated on the Potchefstroom campus of North‐West University, for baseline and follow‐up data collection. For both phases, participants were requested to fast from 22:00 pm on the evening prior to their arrival at the clinic. A maximum of four participants arrived at the clinic at approximately 08:00 on the day of participation, after which all procedures and measurements were reiterated. Participants had the opportunity to ask questions, after which they provided written informed consent. A registered research nurse supervised all procedures and collected fasting blood samples early in the morning. Various measurements and assessments were completed throughout the day (as described later in this study). The participants also received direct feedback on selected measurements and if any health‐related irregularities were identified, participants were referred to a healthcare provider.

### 2.3. Depressive Symptom Assessment

Depressive symptoms were assessed at baseline and follow‐up using the validated nine‐item Patient Health Questionnaire (PHQ‐9), which is based on the diagnostic criteria for MDD outlined in the Diagnostic and Statistical Manual of Mental Disorders, Fourth Edition (DSM‐IV) and retained in the current Fifth Edition (DSM‐5‐TR) [11]. Each item is rated on a 4‐point Likert scale (0 = “not at all” to 3 = “nearly every day”), yielding a total score ranging from 0 to 27, with higher scores indicating more severe depressive symptoms. Internal consistency was evaluated using the nine items using Cronbach’s alpha (*α*) and McDonald’s omega (*ω*). The scale has demonstrated good reliability (*α* = 0.82; *ω* = 0.82). A cut‐off score of ≥10 was used to indicate moderate‐to‐severe depressive symptoms [12], a threshold shown to correlate well with a clinical diagnosis of MDD across adult age groups [13, 14].

### 2.4. Dietary Data

Three 24‐h dietary recall interviews were conducted at baseline by trained fieldworkers. The first interview was conducted on the same day as the other study measurements, and the two follow‐up interviews were conducted on two separate days within the following week, including 1 weekend day to obtain information on weekend food consumption. A standardised dietary collection kit was used, containing visual aids such as pictures, food packages, household measurement tools and food models. Food portion sizes were estimated using plastic food models, household utensils, food packaging materials and a portion‐size photograph book. The South African Medical Research Council (SAMRC) food composition tables [15] were used to code the dietary recalls, and the food quantities manual [16] was employed to convert household measures into grams. When specific food items were unavailable in the tables, they were purchased, weighed and documented for future reference. Final codes and quantities for each dietary record were cross‐checked against the original recall data to ensure accuracy in coding and data capturing. Nutrient analysis of the baseline dietary data was conducted by the SAMRC Biostatistics Unit using South African food composition tables [15]. The individual average intakes of energy and the GBD‐defined dietary exposures were calculated from the three dietary recalls for each participant. These food groups and nutrients as defined by the GBD included fruit, vegetables, legumes, wholegrains, nuts and seeds, milk, red meat, processed meat, sugar‐sweetened beverages, total fibre, calcium and omega‐3 fatty acids (eicosapentaenoic acid [EPA] and docosahexaenoic acid [DHA]) expressed in grams per day (g/day) and polyunsaturated fatty acids (PUFAs) expressed as percentage (%) of total energy (TE) per day. Although the GBD study does not currently include data on UPF and non‐UPF consumption, these variables were incorporated using the NOVA classification system and expressed separately as a % of TE intake [17]. To enhance the practical interpretability of the findings, the units of some of the food groups and nutrients were rescaled to reflect estimated daily consumption based on dietary intake data from South African adults [18–21]. Accordingly, fruit was expressed as increments of 100 g/day, vegetables as 100 g/day, legumes as 25 g/day, wholegrains as 50 g/day, milk as 10 g/day, red meat as 25 g/day, processed meat as 17 g/day and sugar‐sweetened beverages as 50 g/day, while all other nutrients were expressed as 1 g/day, PUFA and UPF each as % of TE.

### 2.5. Demographic and Medical History Questionnaires

A general health questionnaire was used to obtain sociodemographic information, including age, sex, race, total household income, highest level of education and employment status. The Kuppuswamy’s Socioeconomic Status (SES) Scale [22] was adapted to calculate a composite socioeconomic score. The scale comprises three components, namely, the total household income, highest level of education and employment status (SASCO skill level). Participants received a total SES score ranging from 5 to 30 and were subsequently categorised into low (5–18 points), middle (19–24 points) or high (25–30 points) SES, as described previously [23].

### 2.6. Body Composition

Body mass index (BMI) was calculated as body weight in kilograms (obtained with the SECA 813 Electronic Scale, SECA, Hamburg, Germany) divided by the square of body height in metres (obtained using the SECA 213 Portable Stadiometer, SECA, Hamburg, Germany).

### 2.7. Statistical Analyses

Statistical analyses were performed using IBM SPSS Statistics version 30 (IBM Corporation; Armonk, New York, USA) and graphical figures were created using GraphPad Prism version 5.03 (GraphPad Software Inc., CA, USA). All variables were tested for normality by making use of quantile–quantile (Q–Q) plots as well as skewness and kurtosis statistics. Variables that were not normally distributed were natural logarithmic (Ln) transformed to follow parametric assumptions (TE and BMI). Descriptive statistics were computed, with continuous variables reported as means ± standard deviations (SD) and categorical variables as the counts (*n*) and percentages (%). Additionally, independent Student’s *t*‐tests were used to compare participant characteristics and dietary exposures between individuals with and without depression at baseline. Student’s *t*‐tests were also used to compare participants who completed the follow‐up assessment (prior to the exclusion of individuals with missing follow‐up depression data) with those who did not attend the follow‐up assessment (Tables S1 and S2). Homogeneity of variances was assessed using Levene’s test, and the assumptions for the parametric analyses were adequately met. To account for multiple comparisons across the dietary variables (*m* = 15), *p*‐values were adjusted using the Simes procedure [24].

Logistic regression analysis was performed to determine whether dietary consumption of specific food groups and nutrients was associated with increased odds of depression at baseline. Poisson regression analyses were then used to evaluate whether baseline dietary consumption was associated with the risk of incident depression at follow‐up. For each regression analysis, four separate models were fitted as Model 1, an unadjusted model; Model 2, adjusted for sociodemographic covariates (age, sex, race and SES); Model 3, adjusted for TE intake using Willett’s residual method [25]; and Model 4, a fully adjusted model including both sociodemographic covariates and TE intake using Willett’s residual method. More than 90% of participants reported no consumption of legumes, nuts or seeds. Consequently, these food groups were excluded from all regression analyses due to an insufficient number of valid cases. Sensitivity analyses were performed by additionally adjusting for body composition (BMI) in the final fully adjusted models (Model 4). For the regression analyses, the normality of the residuals was assessed to confirm that the assumptions of the regression models were met. *p*‐Values were adjusted for multiple testing using the Simes procedure [24]. For each family of 13 hypotheses, ordered *p*‐values were compared with Simes‐adjusted critical values.

## 3. Results

### 3.1. Baseline Cohort Description

In the total cohort included at baseline (*n* = 1039), the prevalence of depression was 29.45% (*n* = 306). Participant characteristics are presented in Table 1 by depression status. The mean age of the participants was 24.55 years, with a balanced distribution of sex (male: 48.6%, female: 51.4%) and race (Black: 48.6%, White: 51.4%) in the total group. Race distribution differed by depression status, with a higher proportion of Black participants among those with depression (62.4%) and a higher proportion of White participants among those without depression (57.2%). SES also differed by depression status, with 57.5% of individuals with depression were classified as having a low SES, whereas 36.7% of individuals without depression were classified as having a high SES. Overall, the average BMI was 24.13 kg/m^2^. No differences were shown between the groups for BMI. Individuals with depression had a lower TE intake compared to those without depression (mean difference: 579.11 kJ/day).

**Table 1:** Baseline cohort description.

Variables	Total cohort, *n* = 1039	No depression, *n* = 733	Depression, *n* = 306	*p*‐Value
Sociodemographic information				
Age (years)	24.55 (24.36; 24.74)	24.69 (24.47; 24.92)	24.20 (23.85; 24.56)	**0.021**
Sex, *n* (%)	—	—	—	0.185
Male	505 (48.6)	366 (49.9)	139 (45.4)	—
Female	534 (51.4)	367 (50.1)	167 (54.6)	—
Race, *n* (%)	—	—	—	**<0.001**
Black	505 (48.6)	314 (42.8)	191 (62.4)	—
White	534 (51.4)	419 (57.2)	115 (37.6)	—
Socioeconomic status, *n* (%)	—	—	—	**<0.001**
Low	402 (38.7)	226 (30.9)	176 (57.5)	—
Medium	313 (30.2)	237 (32.4)	76 (24.8)	—
High	323 (31.1)	269 (36.7)	54 (17.6)	—
SES score (points)	20.73 (20.36; 21.09)	21.80 (21.38; 22.21)	18.17 (17.51; 18.83)	**<0.001**
Depression				
PHQ‐9 (total score)	7 (0, 27)	5 (0, 9)	14 (10, 27)	**<0.001**
Lifestyle‐related descriptors				
^a^Total energy intake (kJ)	6478.18 (3440.35)	6672.60 (3446.27)	6093.49 (3156.23)	**<0.001**
^a^BMI (kg/m^2^)	24.13 (6.69)	24.21 (6.27)	23.80 (7.85)	0.628

*Note:* The data are presented as mean (95% CI), number of participants (%) or median (±IQR) for ^a^non‐parametric logarithmically transformed variables or mean (minimum to maximum range) for the depression score. Values in bold are regarded as statistically significant (*p* ≤ 0.05).

Abbreviations: BMI, body mass index; PHQ, patient health questionnaire; SES, socioeconomic status.

Table 2 presents the average intake of dietary exposures at baseline. Individuals with depression had a lower intake of fruit (mean difference: 27.5 g/day), vegetables (mean difference: 25 g/day), legumes (mean difference: 13 g/day), whole grains (mean difference: 12.5 g/day), fibre (mean difference: 0.64 g/day), PUFA (mean difference: 2.07 g/day) and calcium (mean difference: 0.12 g/day), as well as higher intake of sugar‐sweetened beverages (mean difference: 33.34 g/day), when compared to individuals without depression (Table 2). After correction for multiple comparisons, only the differences in fruit, vegetable, wholegrain, fibre, PUFA and calcium intake remained.

**Table 2:** Average intake of dietary exposures at baseline.

Dietary exposures	Total cohort	No depression	Depression	*p*‐Value
Fruit intake (g/day) (*n* = 444)	145.26 (150.85)	100.00 (150.00)	72.50 (116.67)	**≤0.001 ^∗^**
Vegetable intake (g/day) (*n* = 842)	131.91 (114.54)	113.33 (105.45)	88.33 (109.68)	**≤0.001 ^∗^**
Legumes intake (g/day) (*n* = 102)	61.94 (86.85)	34.50 (63.25)	21.50 (40.40)	**0.040**
Wholegrain intake (g/day) (*n* = 341)	85.67 (85.71)	62.50 (73.50)	50.00 (52.29)	**0.016 ^∗^**
Nuts and seeds intake (g/day) (*n* = 61)	30.29 (26.23)	20.00 (38.33)	23.33 (17.50)	0.920
Milk intake (g/day) (*n* = 719)	168.44 (158.74)	140.00 (181.75)	110.00 (167.50)	0.109
Red meat intake (g/day) (*n* = 540)	115.03 (96.39)	100.00 (92.50)	89.83 (92.92)	0.205
Processed meat intake (g/day) (*n* = 640)	65.68 (60.78)	46.67 (58.33)	50.00 (63.33)	0.404
Sugar‐sweetened beverages intake (g/day) (*n* = 641)	362.58 (385.94)	333.33 (325.00)	366.67 (358.33)	**0.025**
Fibre intake (g/day) (*n* = 1022)	14.79 (8.00)	13.27 (9.46)	12.63 (7.74)	**0.001 ^∗^**
Calcium intake (g/day) (*n* = 1022)	0.52 (0.36)	0.48 (0.50)	0.36 (0.38)	**≤0.001 ^∗^**
Omega‐3 fatty acid intake (g/day) (*n* = 1022)	168.77 (434.46)	65.67 (126.83)	53.50 (103.52)	0.349
Polyunsaturated fat intake (% of TE) (*n* = 1014)	17.03 (10.94)	15.20 (13.19)	13.13 (11.96)	**0.013 ^∗^**
Ultra‐processed food intake (% of TE) (*n* = 1014)	46.09 (18.15)	45.09 (25.47)	47.20 (23.94)	0.259
Non‐ultra‐processed food intake (% of TE) (*n* = 1021)	54.32 (18.45)	55.22 (25.47)	53.00 (24.14)	0.483

*Note:* The data are presented as mean (SD). *n*‐values represent the number of valid cases per dietary risk factor for the total cohort. Values in bold are regarded as statistically significant (*p* ≤ 0.05). Superscript ( ^∗^) indicates results that remained after adjustment for multiple comparisons.

### 3.2. Cross‐Sectional Analyses

As presented in Table 3, each 1‐g increment in calcium intake was associated with lower odds of depression in Model 1 (odds ratio [OR] = 0.39; 95% CI: 0.25–0.59) and Model 3 (OR = 0.48, 95% CI: 0.31–0.76), but not in the sociodemographic‐adjusted models (Models 2 and 4). Each 50‐g increment in whole grain intake was associated with lower odds of depression in all models: Model 1 (OR = 0.82, 95% CI: 0.67–1.00), Model 2 (OR = 0.78, 95% CI: 0.64–0.95), Model 3 (OR = 0.82, 95% CI: 0.67–1.00) and Model 4 (OR = 0.78, 95% CI: 0.64–0.95). Each 100‐g increment in vegetable intake was associated with lower odds of depression in Model 1 (OR = 0.74, 95% CI: 0.61–0.82), Model 2 (OR = 0.82, 95% CI: 0.67–1.00) and Model 3 (OR = 0.74, 95% CI: 0.61–0.91), but not in Model 4. Fibre intake (each 1‐g increment) was associated with lower odds of depression in Model 1 (OR = 0.97, 95% CI: 0.96–0.99) and Model 2 (OR = 0.98, 95% CI: 0.96–1.00), but not in the models adjusted for TE intake (Models 3 and 4).

**Table 3:** Association between dietary intake and the probability of depression.

Dietary exposure	PHQ > 10		
	OR	95% CI	*p*‐Value
Model 1			
Fruit per 100 g	0.819	0.670; 1.000	0.057
Vegetables per 100 g	0.740	0.606; 0.819	**<0.001 ^∗^**
Wholegrains per 50 g	0.818	0.669; 1.000	**0.033**
Milk per 10 g	1.000	0.990; 1.010	0.946
Red meat per 25 g	0.951	0.882; 1.000	0.064
Processed meat per 17 g	1.017	0.983; 1.070	0.291
Sugar‐sweetened beverages per 50 g	1.000	1.000; 1.051	**0.031**
Fibre per 1 g	0.974	0.957; 0.992	**0.005 ^∗^**
Calcium per 1 g	0.386	0.251; 0.592	**<0.001 ^∗^**
Omega‐3 fatty acids per 1 g	1.000	1.000; 1.000	0.815
PUFA per % of TE	0.995	0.961; 1.030	0.769
UPF per % of TE	1.004	0.996; 1.011	0.345
Non‐UPF per % of TE	0.997	0.990; 1.005	0.483
Model 2			
Fruit per 100 g	0.905	0.740; 1.000	0.144
Vegetables per 100 g	0.819	0.670; 1.000	**0.036**
Wholegrains per 50 g	0.778	0.636; 0.951	**0.019**
Milk per 10 g	1.010	1.000; 1.020	0.194
Red meat per 25 g	0.951	0.905; 1.025	0.218
Processed meat per 17 g	1.052	1.000; 1.107	**0.025**
Sugar‐sweetened beverages per 50 g	1.051	1.000; 1.051	**0.023**
Fibre per 1 g	0.977	0.958; 0.995	**0.015**
Calcium per 1 g	0.743	0.468; 1.180	0.209
Omega‐3 fatty acids per 1 g	1.000	1.000; 1.000	0.599
PUFA per % of TE	0.998	0.963; 1.034	0.902
UPF per % of TE	1.005	0.998; 1.013	0.176
Non‐UPF per % of TE	0.995	0.988; 1.003	0.228
Model 3			
Fruit per 100 g	0.905	0.740; 1.000	0.103
Vegetables per 100 g	0.740	0.606; 0.905	**0.001 ^∗^**
Wholegrains per 50 g	0.818	0.669; 1.000	**0.029**
Milk per 10 g	1.000	0.990; 1.010	0.560
Red meat per 25 g	0.975	0.905; 1.025	0.294
Processed meat per 17 g	1.052	1.000; 1.107	0.056
Sugar‐sweetened beverages per 50 g	1.051	1.000; 1.051	**0.004 ^∗^**
Fibre per 1 g	0.987	0.967; 1.007	0.167
Calcium per 1 g	0.480	0.305; 0.756	**0.002 ^∗^**
Omega‐3 fatty acids per 1 g	1.000	1.000; 1.000	0.984
PUFA per % of TE	0.997	0.963; 1.0032	0.860
UPF per % of TE	1.004	0.997; 1.012	0.296
Non‐UPF per % of TE	0.997	0.990; 1.004	0.397
Model 4			
Fruit per 100 g	0.905	0.740; 1.105	0.191
Vegetables per 100 g	0.819	0.740; 1.000	0.061
Wholegrains per 50 g	0.778	0.636; 0.951	**0.014**
Milk per 10 g	1.010	1.000; 1.020	0.090
Red meat per 25 g	0.975	0.905; 1.025	0.388
Processed meat per 17 g	1.070	1.017; 1.126	**0.007**
Sugar‐sweetened beverages per 50 g	1.051	0.254; 1.051	**0.010**
Fibre per 1 g	0.982	0.961; 1.003	0.100
Calcium per 1 g	0.943	0.565; 1.574	0.822
Omega‐3 fatty acids per 1 g	1.000	1.000; 1.000	0.703
PUFA per % of TE	0.999	0.964; 1.035	0.954
UPF per % of TE	1.006	0.998; 1.013	0.159
Non‐UPF per % of TE	0.995	0.987; 1.003	0.197

*Note:* Model 1, unadjusted. Model 2, adjusted for sociodemographic information (age, sex, ethnicity and socioeconomic status). Model 3, adjusted for total energy intake using Willetts residual method. Model 4, adjusted for sociodemographic information and total energy intake using Willetts residual method. Values in bold are regarded as statistically significant (*p* ≤ 0.05). Superscript ( ^∗^) indicates results that remained after adjustment for multiple comparisons.

Abbreviations: CI, confidence interval; OR, odds ratio; PUFA, polyunsaturated fat; UPF, ultra‐processed food.

Each 17‐g increment in processed meat consumption was associated with higher odds for depression, but only in the models adjusted for sociodemographic information: Model 2 (OR = 1.05, 95% CI: 1.00–1.11) and Model 4 (OR = 1.07, 95% CI: 1.02–1.13). Intake of sugar‐sweetened beverages (each 50‐g increment) was associated with higher odds of depression in all the models: Model 1 (OR = 1.00, 95% CI: 1.00–1.05), Model 2 (OR = 1.05, 95% CI: 1.00–1.05), Model 3 (OR = 1.05, 95% CI: 1.00–1.05) and Model 4 (OR = 1.05, 95% CI: 0.25–1.05).

No associations were observed for fruit, milk, red meat, omega‐3 fatty acids, PUFA, UPF or non‐UPF in any of the models (Table 3). Additional adjustment for BMI in the sensitivity model (Model 4 and BMI) did not affect the results in any of the models, as shown in Table S3. After adjustment for multiple comparisons, associations in Model 1 between vegetables, fibre and calcium and odds for depression remained. In Model 3, associations with vegetables, sugar‐sweetened beverages and calcium remained. In contrast, all associations in Models 2 and 4 were lost, as shown in Table 3.

### 3.3. Longitudinal Analyses

The average follow‐up time in this study was 4.96 years. The retention rate for participants included in this sample from the African‐PREDICT cohort was 58.52%, with 608 of the 1039 baseline participants completing the follow‐up assessment prior to excluding individuals with missing follow‐up depressive symptom data (*n* = 159). To assess the potential for selection bias, baseline characteristics were compared between participants who successfully completed the follow‐up assessment (*n* = 608, prior to the exclusion of individuals with missing follow‐up depression data) and those who did not attend the follow‐up assessment (*n* = 431), as presented in Tables S1 and S2. Participants who completed follow‐up were, on average, approximately 5 months older than those lost to follow‐up (mean age: 24.72 vs. 24.30 years). The majority of participants in both groups were classified as having a low SES. No other significant differences were observed between the two groups.

Among the 551 participants included at follow‐up (Figure 1), 353 did not meet the criteria for depression at either time point. Incident depression was observed in 50 individuals. Remission occurred in 80 participants who met depression criteria at baseline but not at follow‐up, while 68 participants met the criteria at both assessments. The results of the Poisson regression analyses are shown in Figure 2a–d and Table S4. In the total cohort, excluding those who had depression at baseline (*n* = 403), higher sugar‐sweetened beverage intake (per 50‐g increment) was associated with a lower risk of incident depression only in the fully adjusted model: Model 4 (relative risk [RR] = 0.95, 95% CI: 0.90–1.00). Milk consumption (per 10‐g increment) was associated with a lower risk of incident depression in Model 1 (RR = 0.95, 95% CI: 0.91–0.99), Model 3 (RR = 0.94, 95% CI: 0.90–0.98) and Model 4 (RR = 0.95, 95% CI: 0.92–0.99), but not in the sociodemographic‐adjusted model (Model 2). Calcium intake (per 1‐g increment) was associated with a lower risk of incident depression in the models that were not adjusted for sociodemographic information: Model 1 (RR = 0.35, 95% CI: 0.13–0.98) and Model 3 (RR = 0.33, 95% CI: 0.11–0.94).

Figure 2Relative risk of baseline dietary intake predicting incident depression in model 1 (a), model 2 (b), model 3 (c) and model 4 (d). g, gram; PHQ, patient health questionnaire; PUFA, poly‐unsaturated fatty acids; UPF, ultra‐processed foods. Superscript ( ^∗^) indicates results that remained after adjustment for multiple comparisons.

**Figure:**
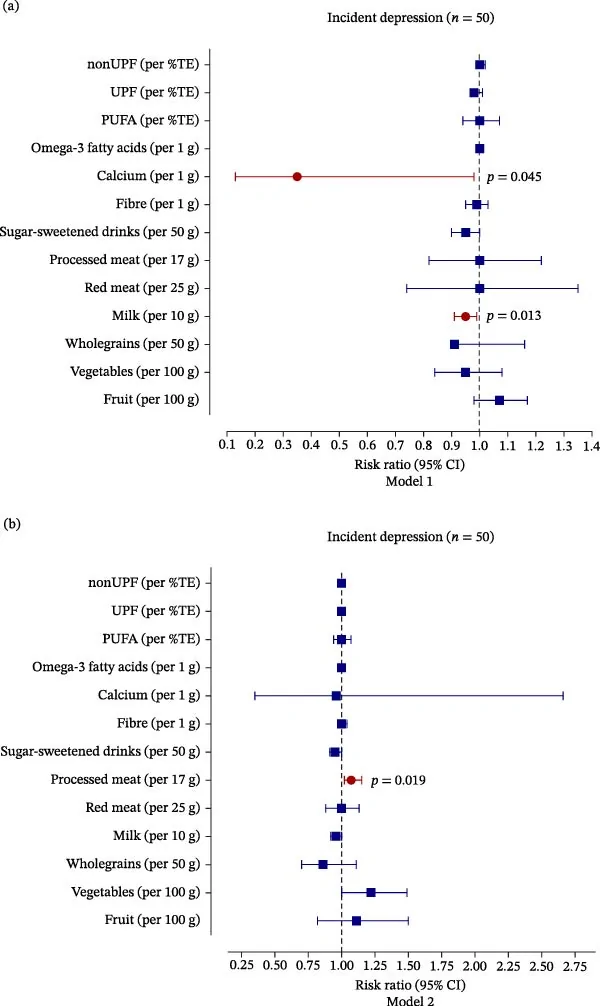

**Figure:**
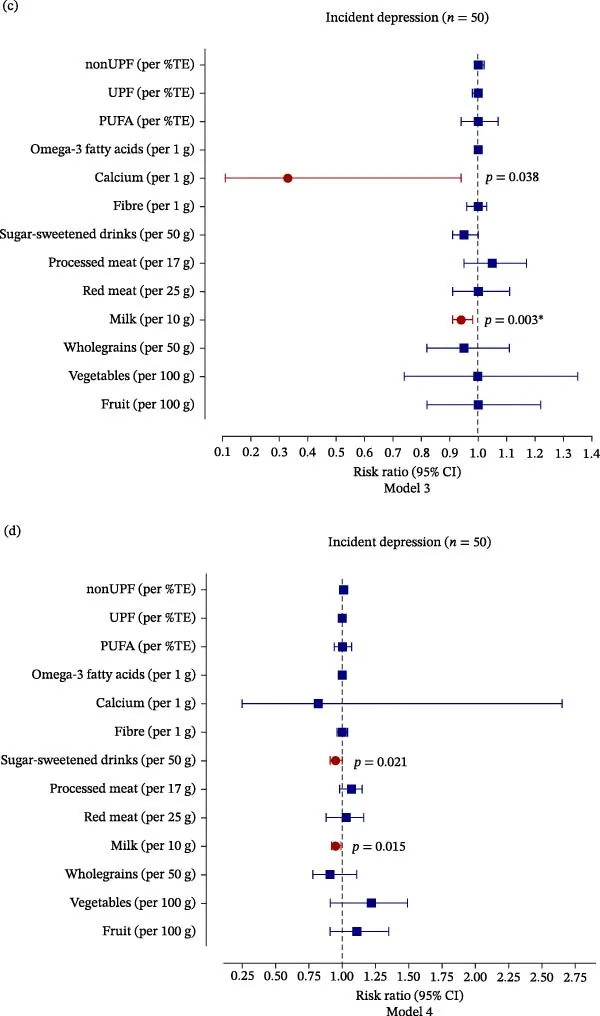

Processed meat consumption (per 17‐g increment) increased the risk of incident depression in Model 2 (RR = 1.07, 95% CI: 1.02–1.15) but not in the unadjusted model (Model 1) or in the models adjusted for TE intake (Models 3 and 4).

Consumption of the other dietary exposures in this model, including fibre, omega‐3 fatty acids, PUFA and UPF, was not associated with incident depression, as shown in Figure 2a–d. Following adjustment for multiple comparisons, all associations in Models 1, 2 and 4 lost statistical significance, while only the association with milk in Model 3 remained significant, as shown in Figure 2c. Additionally, adjusting for BMI in the sensitivity model (adjusting for covariates included in Model 4 and BMI) did not affect the outcome of the results in any of the models (Table S3).

## 4. Discussion

This study investigated the association between dietary intake–defined according to the GBD study, and symptoms of depression in a cohort of young South African adults. In the cross‐sectional analysis, higher intakes of calcium and vegetables were associated with lower odds for depression in the unadjusted models and models adjusted for TE intake. Higher intakes of fibre were associated with lower odds only in the unadjusted analysis. Higher sugar‐sweetened beverage consumption was associated with higher odds for depression, but only after adjustment for TE intake. In the longitudinal analyses, there was some evidence that higher intake of milk may be associated with a lower risk of incident depression from baseline to follow‐up; however, this was only present when TE was adjusted for.

### 4.1. Sugar‐Sweetened Beverages as Risk Factor for Depression

Cross‐sectionally, sugar‐sweetened beverage consumption was associated with higher odds for depression in the models adjusted for TE intake, consistent with previous findings [26–28]. Increased sugar‐sweetened beverage consumption has been linked to reduced dopaminergic brain responses in the posterior midbrain and dorsolateral prefrontal cortex of young adults [29], regions suspected to be implicated in the pathophysiology of depression. These findings appear to be driven by the increased intake of sugar content rather than total caloric‐intake [29], which aligns with our finding that it only became significant after adjusting for TE intake. High‑sugar diets may contribute to depression through multiple biological pathways, including disrupted insulin signalling, hypothalamic–pituitary–adrenal (HPA)‐axis dysregulation, systemic inflammation, impaired neuroplasticity and neurotransmitter function and alterations in the gut–brain axis. Collectively, these effects can interfere with brain regions and signalling systems critical for mood regulation [30].

In contrast to the cross‐sectional findings, longitudinal analyses suggested that sugar‐sweetened beverage consumption at baseline was associated with a lower risk of incident depression over time, which is inconsistent with the vast majority of the literature and lacks biological plausibility. Therefore, it is likely to be either a spurious finding owing to statistical chance, small sample size at follow‐up or methodological limitations, particularly given that the association was observed only in the model adjusted for both TE intake and sociodemographic factors and did not remain significant after correction for multiple comparisons. For example, we did not capture changes in dietary exposures over time to know whether sugar‐sweetened beverage consumption reduced over the study period, which may have helped explain this protective finding. It is plausible that population level reductions in sugar‐sweetened beverage occurred—notably, sugar‐sweetened beverage intake decreased by approximately 33% between 2018 and 2023 [31], a trend also observed in South African children living in the same geographical area as the young adults in our cohort [32]. Such a decline would be consistent with existing evidence suggesting that reductions in sugar‐sweetened beverage intake may have beneficial effects on mental health outcomes. This may particularly be true for young adults, as they were reported to consume more sugar‐sweetened beverages than other age groups [33].

The discrepancy in the direction of associations observed between the cross‐sectional and longitudinal analyses may also reflect reverse causality in the cross‐sectional findings, as causal inferences cannot be drawn from cross‐sectional data alone. In our cohort, individuals with depression reported higher consumption of sugar‐sweetened beverages than those without depression. Although this difference may indicate that individuals with depressive symptoms consumed these beverages more frequently as a potential coping mechanism, the difference was attenuated after correction for multiple comparisons and should be interpreted with caution. Previous findings among young adults have shown that the cross‐sectional association between depressive symptoms and sugar consumption is mediated by emotional dysregulation, including emotional eating and food cravings [34].

### 4.2. The Association Between Processed Meat Consumption and Depression

In the sociodemographic‐adjusted cross‐sectional and longitudinal models, processed meat consumption was positively associated with depression, in line with previous research [35]. Processed meat, usually defined as products made of red meat that has been cured, salted or smoked to extend shelf‐life, is generally high in saturated fat, sodium, nitrites/nitrates and pro‐oxidant compounds such as advanced glycation end‐products (AGEs) [36]. Such a profile may promote inflammation and oxidative stress, thereby increasing the risk of depression. In contrast to a study that found a higher intake of processed meat to increase the risk of developing late‐onset depression in older adults over a 12‐year period [37], this association was not observed in the unadjusted or TE‐adjusted‐only models, suggesting that sociodemographic factors may confound the relationship. Indeed, previous studies in South Africa have shown that processed meat consumption is higher among men, Black individuals, those with higher SES, and young adults [38]. Furthermore, the findings with processed meat consumption were no longer significant after multiple comparison corrections and therefore must be interpreted with caution.

### 4.3. Calcium Intake and Milk Consumption as Potential Protective Factors for Depression

Several micronutrient deficiencies contribute to the pathophysiology of depression. Consistent with our cross‐sectional and longitudinal findings, several studies have reported an inverse association between dietary calcium intake and the prevalence of depressive symptoms [39–41]. Calcium is essential for neurotransmission through its involvement in various types of calcium channels that regulate presynaptic neurotransmitter release, postsynaptic signalling and synaptic plasticity [42]. Disruptions in long‐term synaptic plasticity, particularly in the prefrontal cortex and hippocampus, have been implicated in depression [43]. Inadequate calcium intake may, therefore, impair synaptic transmission and plasticity, contributing to the depressive pathology. Calcium intake was lower in those with depression compared to individuals without depression at baseline, underscoring the potential importance of calcium deficiencies. Sociodemographic context appears particularly important, as these findings were not observed in the models adjusted for sociodemographic factors, indicating that the associations may reflect influences of specific sexes, races, socioeconomic groups or interactions among these factors. In our study, participants with depression were predominantly Black and had a low SES. Furthermore, previous African‐PREDICT findings showed that black individuals had lower calcium intakes than White individuals, although neither group met the estimated average calcium requirements, with more than 50% of the entire cohort across all SES groups not meeting these levels [44].

Similarly, milk consumption was associated with a lower risk of incident depression in the longitudinal analyses. Although the specific mechanisms underlying the inverse findings remain unclear, as reflected by the contradicting results reported on the relationship between depression and milk intake in the literature [45], the nutritional composition of milk may be relevant. Milk provides a substantial source of dietary calcium, supporting both calcium intake and levels. The chief protein in mammalian milk, casein, supports calcium absorption and provides amino acids important for neurotransmitter synthesis, and has been shown, in combination with GABA, to reduce depressive‐like behaviour in animal models [46, 47]. Fat content may also be relevant as low‐fat varieties, such as semi‐skimmed and skimmed milk, are associated with a lower risk of depression, whereas whole milk may increase the risk [48, 49]. However, we did not observe these findings in our cross‐sectional analyses, suggesting that the effects of inadequate milk intake on depressive symptoms may only become noticeable after a longer period of time, with limited measurable effects detectable in the short term. Indeed, young adulthood may represent a key developmental period during which the effects of inadequate milk intake on depression are not immediately observable but emerge over time. Consistent with this, no differences in milk consumption were observed between individuals with and without depression at baseline, which may explain why associations were only observed in the longitudinal analyses. Longitudinal assessment of dietary changes would therefore further clarify these inverse associations.

### 4.4. The Inverse Associations Between Wholegrain, Fibre and Vegetable Consumption With Depression

We observed inverse cross‐sectional associations between depression and intake of wholegrains, dietary fibre and vegetables, partially supporting previous evidence that higher consumption of these foods may be protective against depressive symptoms [50–53]. However, none of these associations were observed in the longitudinal analyses, and most were no longer significant after adjustment for TE intake or correction for multiple comparisons. It is possible that the cross‐sectional associations may reflect reverse causality as individuals with depression exhibited lower consumption of wholegrains, fibre and vegetables when compared to those without or residual confounding rather than a sustained effect over time. Overall, these findings highlight the need for cautious interpretation and underscore the challenges of disentangling dietary influences on depression in young adults, where evidence remains inconsistent.

### 4.5. Absence of Associations With UPF Consumption

Various studies have reported that UPF consumption increases the risk of depression [54, 55]. The absence of an association in our study remains unclear but does not necessarily indicate that no relationship exists. Rather, it suggests that the relationship between UPF consumption and depression may depend on the characteristics of the study population and the broader dietary context. Indeed, heterogeneous associations have been reported between different UPF subtypes and health outcomes [56]. This variability has also been observed in relation to depression. A recent systematic review and meta‐analysis reported an overall positive association between UPF consumption and depression; however, the analyses were characterised by substantial heterogeneity, reflecting differences in assessment tools and study populations [57].

For instance, associations between UPF consumption and psychiatric symptoms were observed when using the Kessler Psychological Distress Scale [58] but not when using the Depression Anxiety Stress Scale [59] or a Self‐Reporting Questionnaire [60]. Regarding the tools used to assess food consumption, UPF consumption was significantly associated with a higher risk of depression but not anxiety in studies that used food frequency questionnaires compared to those employing other dietary recall methods [54]. In this study, we utilised the NOVA classification system to define UPF consumption. Although widely used, the NOVA classification has been reported to result in inconsistencies in the categorisation of foods across different contexts [56], which should be considered when comparing findings across study settings. There also seems to be a misalignment between the processing level and nutritional quality reported by the NOVA classification [61], as it seems to be driven largely by nutrient content rather than processing itself [62]. UPF consumption, as classified using the NOVA classification system, has been shown to contribute disproportionately to TE intake, particularly among South African individuals with the highest UPF consumption [63]. Compared with low UPF consumers, high UPF consumers had a greater TE intake and higher intakes of sodium, sugar, fat and processed meat. However, they were also more likely to meet the World Health Organization (WHO) recommendations for nuts and seeds, whole grains and fibre, while no differences were observed in fruit and vegetable intake. These findings suggest that the NOVA classification may not adequately capture overall dietary quality, as individuals with high UPF consumption may simultaneously consume foods of high diet quality. Consequently, UPF consumption classified according to NOVA may not be independent of the overall dietary pattern, which could partly explain the null findings observed in our study.

### 4.6. Strengths and Limitations

This study offers valuable insights into the relationship between dietary intake and depression, laying a foundation for future longitudinal research to assess whether dietary interventions could improve depressive symptoms. However, certain limitations must be acknowledged. The African‐PREDICT study included a screened cohort of apparently healthy young adults without overt CVD, all residing within a specific geographical area. As a result, the findings may not be generalisable to the broader South African population. The relatively high attrition observed in this study is a limitation; however, it is a recognised challenge in longitudinal cohort studies conducted in African settings and is often driven by high levels of residential mobility and migration [64, 65]. While validated tools were used to assess the presence and severity of depression, the use of a structured clinical interview would have strengthened the reliability of the findings by capturing the symptom duration beyond the preceding 2 weeks.

## 5. Conclusion

Milk consumption at baseline was associated with a lower risk of incident depression at follow‐up. In addition, depression was associated positively with sugar‐sweetened beverages and inversely with fibre, vegetables and calcium in cross‐sectional analyses. However, while these dietary factors were associated with depression in the unadjusted and TE‐adjusted analyses, these associations were attenuated following adjustment for sociodemographic information. These findings highlight the importance of considering the sociodemographic context when incorporating dietary considerations into mental health prevention strategies. Future research should further explore the complex interplay between diet, sociodemographic factors and mental health in young adults.

## Author Contributions

Esmé Jansen van Vuren contributed to data analysis and interpretation, writing – original draft and writing – review and editing. Adrienne O’Neil, Deborah N. Ashtree, Melissa M. Lane and Rebecca Orr contributed to conceptualisation, methodology ‐ developing the methods for the GLAD project, project administration ‐ management and coordination of the GLAD project, resources ‐ provision of study materials, including the data analysis code and materials of the GLAD project and writing – review and editing. Marlien Pieters contributed to writing – review and editing. Tertia van Zyl conducted sample and data analysis and contributed to writing – review and editing.

## Funding

The research funded in this manuscript is part of an ongoing research project financially supported by the South African Medical Research Council (SAMRC) with funds from National Treasury under its Economic Competitiveness and Support Package and SAMRC Extramural Research Unit Funding (Grant SAMRC‐RFA‐EMU‐10‐2020); the South African Research Chairs Initiative (SARChI) of the Department of Science and Technology and National Research Foundation (NRF) of South Africa (Grant GUN 86895); the SAMRC, with funds received from the South African National Department of Health, GlaxoSmithKline R&D (Africa Non‐Communicable Disease Open Lab Grant), the UK Medical Research Council and with funds from the UK Government’s Newton Fund; and corporate social investment grants from Pfizer (South Africa), Boehringer‐Ingelheim (South Africa), Novartis (South Africa), the Medi Clinic Hospital Group (South Africa) and in kind contributions of Roche Diagnostics (South Africa). The GLAD project is funded by the National Health and Medical Research Council Emerging Leader 2 Fellowship (Grant 2009295 [Adrienne O’Neil]).

## Disclosure

The opinions, methods and conclusions reported in this paper are those of the authors and are independent from the funding sources. Accordingly, the NRF accepts no liability for the opinions, findings, conclusions or recommendations expressed in this material. This manuscript has been prepared in accordance with the requirements of the GLAD Taskforce, as part of a global collaborative project to inform the Global Burden of Diseases, Injuries, and Risk Factors Study. All authors read and approved the final manuscript.

## Conflicts of Interest

Melissa M. Lane is a member and former Secretary of the Melbourne Branch Committee of the Nutrition Society of Australia (unpaid). She has received travel funding support from the International Society for Nutritional Psychiatry Research, the Nutrition Society of Australia, the Australasian Society of Lifestyle Medicine, and the Gut Brain Congress. She is also an associate investigator for the MicroFit Study, an investigator‐led randomised controlled trial examining the effects of diets with varying levels of industrial processing on gut microbiome composition, partially funded by Be Fit Food (payment received by the Food & Mood Centre, Deakin University). The other authors declare no conflicts of interest.

## Supporting Information

Additional supporting information can be found online in the Supporting Information section.

## Supporting information


**Supporting Information** Table S1: Baseline characteristics of participants who completed the follow‐up assessment compared with those who were enrolled at baseline but did not participate in the follow‐up phase. Data is presented as mean (95% CI), number of participants (%), or median (±IQR) for  ^∗^non‐parametric logarithmically transformed variables or mean (minimum to maximum range) for the depression score. Values in bold are regarded as statistically significant (*p* ≤ 0.05). Abbreviations: BMI, body mass index; PHQ, patient health questionnaire, SES, socioeconomic status. Table S2: Average intake of dietary exposures at baseline of participants who completed the follow‐up assessment compared with those who were enrolled at baseline but did not participate in the follow‐up phase. Data is presented as mean (SD). *n*‐values represent the number of valid cases per dietary risk factor for the total cohort. Values in bold are regarded as statistically significant (*p* ≤ 0.05). None of these results remained after adjustment for multiple comparisons. Table S3: Sensitivity analyses of associations between dietary intake and depression after additional adjustment for BMI. Adjusted for body mass index in addition to sociodemographic information and total energy intake using Willetts residual method. Abbreviations: BMI, body mass index; CI, confidence interval; OR, odds ratio; PUFA, polyunsaturated fat; UPF, ultra‐processed food. Values in bold are regarded as statistically significant (*p* ≤ 0.05). Superscript ( ^∗^) indicates results that remained after adjustment for multiple comparisons. Table S4: Association between dietary intake and the relative risk of incident depression between baseline and follow‐up. Model 1 = unadjusted; Model 2 = adjusted for sociodemographic information (age, sex, ethnicity and socio‐economic status); Model 3 = adjusted for total energy intake using Willetts residual method; and Model 4 = adjusted for sociodemographic information and total energy intake using Willetts residual method. Abbreviation CI, confidence interval; PUFA, polyunsaturated fat; RR, relative risk; UPF, ultra‐processed food. Values in bold are regarded as statistically significant (*p* ≤ 0.05). Superscript ( ^∗^) indicates results that remained after adjustment for multiple comparisons.

## Data Availability

The datasets generated and/or analysed during the current study are available from the corresponding author upon reasonable request.

## References

[1] Mao Q. S. , Guo Y. X. , Tian X. L. , Zhao H. L. , and Kong Y. Z. , Global Burden of Mental Disorders in 204 Countries and Territories Results From the Global Burden of Disease Study 2021, World Journal of Psychiatry. (2025) 15, no. 8, 10.5498/wjp.v15.i8.106887, 106887. PMC12362664 40837810

[2] Ferrari A. , Santomauro D. , and Mantilla Herrera A. , et al.Global, Regional, and National Burden of 12 Mental Disorders in 204 Countries and Territories, 1990–2019: A Systematic Analysis for the Global Burden of Disease Study 2019, The Lancet Psychiatry. (2022) 9, no. 2, 137–150, 10.1016/S2215-0366(21)00395-3. PMC8776563 35026139

[3] Varma P. , Junge M. , Meaklim H. , and Jackson M. L. , Younger People Are More Vulnerable to Stress, Anxiety and Depression During COVID-19 Pandemic: A Global Cross-Sectional Survey, Progress in Neuro-Psychopharmacology and Biological Psychiatry. (2021) 109, 10.1016/j.pnpbp.2020.110236, 110236. PMC7834119 33373680

[4] Jacka F. N. , Cherbuin N. , Anstey K. J. , Butterworth P. , and Chavatte-Palmer P. , Dietary Patterns and Depressive Symptoms Over Time: Examining the Relationships With Socioeconomic Position, Health Behaviours and Cardiovascular Risk, PLoS ONE. (2014) 9, no. 1, 10.1371/journal.pone.0087657. PMC3906192 24489946

[5] Opie R. S. , O’Neil A. , Itsiopoulos C. , and Jacka F. N. , The Impact of Whole-of-Diet Interventions on Depression and Anxiety: A Systematic Review of Randomised Controlled Trials, Public Health Nutrition. (2015) 18, no. 11, 2074–2093, 10.1017/S1368980014002614. PMC10271872 25465596

[6] Lai J. S. , Hiles S. , Bisquera A. , Hure A. J. , McEvoy M. , and Attia J. , A Systematic Review and Meta-Analysis of Dietary Patterns and Depression in Community-Dwelling Adults, The American Journal of Clinical Nutrition. (2014) 99, no. 1, 181–197, 10.3945/ajcn.113.069880. 24196402

[7] Ashtree D. N. , Orr R. , and Lane M. M. , et al.Estimating the Burden of Common Mental Disorders Attributable to Lifestyle Factors: Protocol for the Global Burden of Disease Lifestyle and Mental Disorder (GLAD) Project, JMIR Research Protocols. (2025) 14, no. 1, 10.2196/65576. PMC11953606 40085831

[8] Craig A. , Rochat T. , and Naicker S. N. , et al.The Prevalence of Probable Depression and Probable Anxiety, and Associations With Adverse Childhood Experiences and Socio-Demographics: A National Survey in South Africa, Frontiers in Public Health. (2022) 10, 10.3389/fpubh.2022.986531, 986531. PMC9650309 36388391

[9] Popkin B. M. and Ng S. W. , The Nutrition Transition to a Stage of High Obesity and Noncommunicable Disease Prevalence Dominated by Ultra-Processed Foods Is Not Inevitable, Obesity Reviews. (2022) 23, no. 1, 10.1111/obr.13366. PMC8639733 34632692

[10] Schutte A. E. , Gona P. N. , and Delles C. , et al.The African Prospective Study on the Early Detection and Identification of Cardiovascular Disease and Hypertension (African-PREDICT): Design, Recruitment and Initial Examination, European Journal of Preventive Cardiology. (2019) 26, no. 5, 458–470, 10.1177/2047487318822354. PMC6423686 30681377

[11] American Psychiatric Association , Diagnostic and Statistical Manual of Mental Disorders, 2022, Fifth edition, American Psychiatric Association Publishing, Washington, DC, USA, text rev. (DSM-5-TR).

[12] Kroenke K. , Spitzer R. L. , and Williams J. B. W. , The PHQ-9: Validity of a Brief Depression Severity Measure, Journal of General Internal Medicine. (2001) 16, no. 9, 606–613, 10.1046/j.1525-1497.2001.016009606.x. PMC1495268 11556941

[13] Moriarty A. S. , Gilbody S. , McMillan D. , and Manea L. , Screening and Case Finding for Major Depressive Disorder Using the Patient Health Questionnaire (PHQ-9): A Meta-Analysis, General Hospital Psychiatry. (2015) 37, no. 6, 567–576, 10.1016/j.genhosppsych.2015.06.012. 26195347

[14] Negeri Z. F. , Levis B. , and Sun Y. , et al.Accuracy of the Patient Health Questionnaire-9 for Screening to Detect Major Depression: Updated Systematic Review and Individual Participant Data Meta-Analysis, BMJ. (2021) 375, 10.1136/bmj.n2183, n2183. PMC8491108 34610915

[15] Wolmarans P. , Danster N. , Dalton A. , Rossouw K. , and Schönfeldt H. , Condensed Food Composition Tables for South Africa, 2010, Medical Research Council, Cape Town, 1–126.

[16] Langenhoven M. , Conradie P. , Wolmarans P. , and Faber M. , MRC Food Quantities Manual, 1991, South African Medical Research Council, Cape Town.

[17] Monteiro C. A. , Cannon G. , and Levy R. B. , et al.Ultra-Processed Foods: What They Are and How to Identify Them, Public Health Nutrition. (2019) 22, no. 5, 936–941, 10.1017/S1368980018003762. PMC10260459 30744710

[18] Pretorius B. , Muller C. , and Schönfeldt H. C. , Nutrient Value and Density of South African Processed Meat, Journal of Food Composition and Analysis. (2025) 143, 10.1016/j.jfca.2025.107476, 107476.

[19] Miller V. , Reedy J. , and Cudhea F. , et al.Global, Regional, and National Consumption of Animal-Source Foods Between 1990 and 2018: Findings From the Global Dietary Database, The Lancet Planetary Health. (2022) 6, no. 3, e243–e256, 10.1016/S2542-5196(21)00352-1. PMC8926870 35278390

[20] Mchiza Z. J. , Steyn N. P. , and Hill J. , et al.A Review of Dietary Surveys in the Adult South African Population From 2000 to 2015, Nutrients. (2015) 7, no. 9, 8227–8250, 10.3390/nu7095389. PMC4586583 26404371

[21] Report G. N. , Country Nutrition Profiles: South Africa, https://globalnutritionreport.org/resources/nutrition-profiles/africa/southern-africa/south-africa/.

[22] Patro B. K. , Jeyashree K. , and Gupta P. K. , Kuppuswamy’s Socioeconomic Status Scale 2010—The Need for Periodic Revision, The Indian Journal of Pediatrics. (2012) 79, no. 3, 395–396, 10.1007/s12098-011-0517-7. 21761123

[23] Strauss-Kruger M. , Pieters M. , and van Zyl T. , et al.Socioeconomic Differences in Nutrient Intake, Metabolite Profiles, and Blood Pressure: The African-PREDICT Study, Journal of Nutrition. (2025) 156, no. 2, 10.1016/j.tjnut.2025.101306, 101306. 41482232

[24] Simes R. J. , An Improved Bonferroni Procedure for Multiple Tests of Significance, Biometrika. (1986) 73, no. 3, 751–754, 10.1093/biomet/73.3.751.

[25] Willett W. C. , Howe G. R. , and Kushi L. H. , Adjustment for Total Energy Intake in Epidemiologic Studies, The American Journal of Clinical Nutrition. (1997) 65, no. 4, 1220S–1228S, 10.1093/ajcn/65.4.1220S. 9094926

[26] Hu D. , Cheng L. , and Jiang W. , Sugar-Sweetened Beverages Consumption and the Risk of Depression: A Meta-Analysis of Observational Studies, Journal of Affective Disorders. (2019) 245, 348–355, 10.1016/j.jad.2018.11.015. 30419536

[27] Guo X. , Park Y. , and Freedman N. D. , et al.Sweetened Beverages, Coffee, and Tea and Depression Risk Among Older US Adults, PLoS ONE. (2014) 9, no. 4, 10.1371/journal.pone.0094715. PMC3990543 24743309

[28] Kim J. , Hong C. , and Lee G. , Association Between Sugar-Sweetened Beverage Consumption and Depression and Suicidal Ideation Among Korean Adults: A Cross-Sectional Study From the 2014 and 2016 Korean National Health and Nutrition Examination Survey (KNHANES), Nutrition Research and Practice. (2022) 16, no. 2, 194–204, 10.4162/nrp.2022.16.2.194. PMC8971820 35392528

[29] Papantoni A. and Burger K. , Increased Consumption of Sugar in Beverages Is Associated With Blunted Dopaminergic Brain Response to High Sugar Taste, Current Developments in Nutrition. (2021) 5, 10.1093/cdn/nzab049_027, 914.

[30] Reis D. J. , Ilardi S. S. , Namekata M. S. , Wing E. K. , and Fowler C. H. , The Depressogenic Potential of Added Dietary Sugars, Medical Hypotheses. (2020) 134, 10.1016/j.mehy.2019.109421, 109421. 31634771

[31] Cejka T. , Piek M. , and Waseem M. , Shifting Sweetness: Impacts of South Africa’s Health Promotion Levy on Sugar-Sweetened Beverages, 2025, WIDER Working Paper.

[32] Kruger H. S. , van Zyl T. , Monyeki M. A. , Ricci C. , and Kruger R. , Decreased Frequency of Sugar-Sweetened Beverages Intake Among Young Children Following the Implementation of the Health Promotion Levy in South Africa, Public Health Nutrition. (2025) 28, no. 1, 10.1017/S1368980024002623. PMC11822614 39764638

[33] Lara-Castor L. , Micha R. , and Cudhea F. , et al.Sugar-Sweetened Beverage Intakes Among Adults Between 1990 and 2018 in 185 Countries, Nature Communications. (2023) 14, no. 1, 10.1038/s41467-023-41269-8, 5957. PMC10614169 37788998

[34] Graybeal A. J. , Stavres J. , and Swafford S. H. , et al.The Associations Between Depression and Sugar Consumption Are Mediated by Emotional Eating and Craving Control in Multi-Ethnic Young Adults, Healthcare. (2024) 12, no. 19, 1944. 10.3390/healthcare12191944 PMC11475145 39408124

[35] Nucci D. , Fatigoni C. , Amerio A. , Odone A. , and Gianfredi V. , Red and Processed Meat Consumption and Risk of Depression: A Systematic Review and Meta-Analysis, International Journal of Environmental Research and Public Health. (2020) 17, no. 18, 10.3390/ijerph17186686, 6686. PMC7559491 32937855

[36] Rohrmann S. and Linseisen J. , Processed Meat: The Real Villain?, Proceedings of the Nutrition Society. (2016) 75, no. 3, 233–241, 10.1017/S0029665115004255. 26621069

[37] Lampignano L. , Sardone R. , and D’Urso F. , et al.Processed Meat Consumption and the Risk of Incident Late-Onset Depression: A 12-Year Follow-up of the Salus in Apulia Study, Age and Ageing. (2022) 51, no. 2, 10.1093/ageing/afab257, afab257. 35134842

[38] Godbharle S. , Kesa H. , Jeyakumar A. , and Shambharkar P. , Socio-Demographic and Economic Factors Associated With the Consumption of Processed Foods in South Africa–Evidence From Demographic and Health Survey VII, Public Health. (2024) 226, 190–198, 10.1016/j.puhe.2023.11.018. 38071952

[39] Miki T. , Kochi T. , and Eguchi M. , et al.Dietary Intake of Minerals in Relation to Depressive Symptoms in Japanese Employees: The Furukawa Nutrition and Health Study, Nutrition. (2015) 31, no. 5, 686–690, 10.1016/j.nut.2014.11.002. 25837213

[40] Alkhatatbeh M. J. , Khwaileh H. N. , and Abdul-Razzak K. K. , High Prevalence of Low Dairy Calcium Intake and Association With Insomnia, Anxiety, Depression and Musculoskeletal Pain in University Students From Jordan, Public Health Nutrition. (2021) 24, no. 7, 1778–1786, 10.1017/S1368980020002888. PMC10195488 32830623

[41] Shen X. , Gu X. , Liu Y.-Y. , Yang L. , Zheng M. , and Jiang L. , Association Between Dietary Calcium and Depression Among American Adults: National Health and Nutrition Examination Survey, Frontiers in Nutrition. (2023) 10, 10.3389/fnut.2023.1042522, 1042522. PMC9948022 36845060

[42] Nanou E. and Catterall W. A. , Calcium Channels, Synaptic Plasticity, and Neuropsychiatric Disease, Neuron. (2018) 98, no. 3, 466–481, 10.1016/j.neuron.2018.03.017. 29723500

[43] Marsden W. N. , Synaptic Plasticity in Depression: Molecular, Cellular and Functional Correlates, Progress in Neuro-Psychopharmacology and Biological Psychiatry. (2013) 43, 168–184, 10.1016/j.pnpbp.2012.12.012. 23268191

[44] Visser M. , Jordaan C. , Wentzel-Viljoen E. , Schutte A. E. , and Van Zyl T. , Nutrient Intake of Young South African Adults From the Baseline of the African-PREDICT Cohort Study, Public Health Nutrition. (2024) 27, no. 1, 10.1017/S1368980024002076. PMC11705013 39468884

[45] Hockey M. , McGuinness A. J. , Marx W. , Rocks T. , Jacka F. N. , and Ruusunen A. , Is Dairy Consumption Associated With Depressive Symptoms or Disorders in Adults? a Systematic Review of Observational Studies, Critical Reviews in Food Science and Nutrition. (2020) 60, no. 21, 3653–3668, 10.1080/10408398.2019.1703641. 31868529

[46] Bhat M. Y. , Dar T. A. , and Singh L. R. , Gigli I. , Casein Proteins: Structural and Functional Aspects, Milk Proteins-From Structure to Biological Properties and Health Aspects, 2016, IntechOpen.

[47] Cai L. , Tao Q. , Li W. , Zhu X. , and Cui C. , The Anti-Anxiety/Depression Effect of a Combined Complex of Casein Hydrolysate and γ-Aminobutyric Acid on C57BL/6 Mice, Frontiers in Nutrition. (2022) 9, 10.3389/fnut.2022.971853, 971853. PMC9554304 36245498

[48] Wu C. , Liu Y. , and Lai Y. , et al.Association of Different Types of Milk With Depression and Anxiety: A Prospective Cohort Study and Mendelian Randomization Analysis, Frontiers in Nutrition. (2024) 11, 10.3389/fnut.2024.1435435, 1435435. PMC11656347 39703337

[49] Sun J. , Wang W. , and Zhang D. , Associations of Different Types of Dairy Intakes With Depressive Symptoms in Adults, Journal of Affective Disorders. (2020) 274, 326–333, 10.1016/j.jad.2020.05.095. 32469823

[50] Wu H. , Zhang S. , and Meng G. , et al.The Consumption of Wholegrain Is Related to Depressive Symptoms Among Chinese Adults: A Cross-Sectional Study, European Journal of Clinical Nutrition. (2022) 76, no. 1, 126–133, 10.1038/s41430-021-00917-2. 33931771

[51] Ross A. B. , Shertukde S. P. , Staffier K. L. , Chung M. , Jacques P. F. , and McKeown N. M. , The Relationship Between Whole-Grain Intake and Measures of Cognitive Decline, Mood, and Anxiety—A Systematic Review, Advances in Nutrition. (2023) 14, no. 4, 652–670, 10.1016/j.advnut.2023.04.003. PMC10334137 37085091

[52] Saghafian F. , Hajishafiee M. , Rouhani P. , and Saneei P. , Dietary Fiber Intake, Depression, and Anxiety: A Systematic Review and Meta-Analysis of Epidemiologic Studies, Nutritional Neuroscience. (2023) 26, no. 2, 108–126, 10.1080/1028415X.2021.2020403. 36692989

[53] Dharmayani P. N. A. , Juergens M. , Allman-Farinelli M. , and Mihrshahi S. , Association Between Fruit and Vegetable Consumption and Depression Symptoms in Young People and Adults Aged 15–45: A Systematic Review of Cohort Studies, International Journal of Environmental Research and Public Health. (2021) 18, no. 2, 10.3390/ijerph18020780, 780. PMC7831325 33477603

[54] Mazloomi S. N. , Talebi S. , and Mehrabani S. , et al.The Association of Ultra-Processed Food Consumption With Adult Mental Health Disorders: A Systematic Review and Dose-Response Meta-Analysis of 260,385 Participants, Nutritional Neuroscience. (2023) 26, no. 10, 913–931, 10.1080/1028415X.2022.2110188. 36094005

[55] Sun M. , He Q. , and Li G. , et al.Association of Ultra-Processed Food Consumption With Incident Depression and Anxiety: A Population-Based Cohort Study, Food & Function. (2023) 14, no. 16, 7631–7641, 10.1039/D3FO01120H. 37534433

[56] Louie J. C. Y. , Are all Ultra-Processed Foods Bad? A Critical Review of the NOVA Classification System, Proceedings of the Nutrition Society. (2025) 1–9. 10.1017/S0029665125100645 40757421

[57] Freres S. C. , Lobo V. S. , Lopatiuk C. , and de Lucena H. C. A. , Association Between Ultra-Processed Food Consumption and Risk of Depressive Symptoms in Adults: A Systematic Review and Meta-Analysis, Journal of Advances in Medicine and Medical Research. (2025) 37, no. 12, 376–388, 10.9734/jammr/2025/v37i126025.

[58] Lane M. M. , Lotfaliany M. , and Hodge A. M. , et al.High Ultra-Processed Food Consumption Is Associated With Elevated Psychological Distress as an Indicator of Depression in Adults From the Melbourne Collaborative Cohort Study, Journal of Affective Disorders. (2023) 335, 57–66, 10.1016/j.jad.2023.04.124. 37149054

[59] José M. E. R. , Ramos I. E. C. , de Sousa T. M. , and Canella D. S. , Food Consumption Associated With Depression, Anxiety and Stress in Students Entering a Public University, Journal of Nutritional Science. (2025) 14, 10.1017/jns.2024.90. PMC11811848 39943931

[60] Werneck A. O. , Costa C. S. , and Horta B. , et al.Prospective Association Between Ultra-Processed Food Consumption and Incidence of Elevated Symptoms of Common Mental Disorders, Journal of Affective Disorders. (2022) 312, 78–85, 10.1016/j.jad.2022.06.007. 35691417

[61] Abreu S. and Liz Martins M. , Cross-Classification Analysis of Food Products Based on Nutritional Quality and Degree of Processing, Nutrients. (2023) 15, no. 14, 10.3390/nu15143117, 3117. PMC10383862 37513535

[62] Drewnowski A. , Gupta S. , and Darmon N. , An Overlap Between “Ultraprocessed” Foods and the Preexisting Nutrient Rich Foods Index?, Nutrition Today. (2020) 55, no. 2, 75–81, 10.1097/NT.0000000000000400. PMC10659579 37987022

[63] Frank T. , Ng S. W. , Lowery C. M. , Thow A.-M. , and Swart E. C. , Dietary Intake of Low-Income Adults in South Africa: Ultra-Processed Food Consumption a Cause for Concern, Public Health Nutrition. (2024) 27, no. 1, 10.1017/S1368980023002811. PMC10882538 38204376

[64] Olumide A. O. , Adebayo E. S. , and Fonn S. , Factors Associated With Attrition in a Longitudinal Study of Health Risk Behaviours and Conditions Among Adolescents in Ibadan, Nigeria, PLoS ONE. (2025) 20, no. 4, 10.1371/journal.pone.0320150. PMC11970656 40184358

[65] Norris S. A. , Richter L. M. , and Fleetwood S. A. , Panel Studies in Developing Countries: Case Analysis of Sample Attrition Over the Past 16 Years Within the Birth to Twenty Cohort in Johannesburg, South Africa, Journal of International Development. (2007) 19, no. 8, 1143–1150, 10.1002/jid.1390.

