# Supplementary material for: Cross‐Sectional and Longitudinal Associations Between Dietary Intake and Depressive Symptoms in Young South African Adults: The African‐PREDICT Study
Source: Depress Anxiety. 2026 Sep 29;2026:2938819. doi: 10.1155/da/2938819 (PMC13624545; doi:10.1155/da/2938819)
Supplement: Supplementary file 1 [file DA-2026-2938819-s001.docx]

**Cross-sectional and longitudinal associations between dietary intake and depressive symptoms in young South African adults: The African-PREDICT study**

*Running title: Nutrition and Mental Health*

Esmé Jansen van Vuren^a,b*^, Adrienne O’Neil^c^, Deborah N Ashtree^c^, Melissa M Lane^c^, Rebecca Orr^c^, Marlien Pieters^b,d^, Tertia van Zyl^b,d^

^a^ Hypertension in Africa Research Team (HART), North-West University, Potchefstroom, South Africa

^b^ South African Medical Research Council: Extramural Unit for Hypertension and Cardiovascular Disease, North-West University, Potchefstroom, South Africa

^c^ IMPACT (the Institute for Mental and Physical Health and Clinical Translation), Food & Mood Centre, Deakin University, Geelong, Australia

^d^ Centre of Excellence for Nutrition, North-West University, Potchefstroom, South Africa

**Supplementary information**

**Supplementary Table 1. Baseline characteristics of participants who completed the follow-up assessment compared with those who were enrolled at baseline but did not participate in the follow-up phase.**

| **Variables** | **Completed follow-up assessment**  ***n* = 608** | **Did not complete follow-up assessment**  ***n* = 431** | ***p*-value** |
| --- | --- | --- | --- |
| *Sociodemographic information* |  |  |  |
| Age, years | 24.72 (24.48; 24.97) | 24.30 (24.01; 24.59) | **0.031** |
| Sex, *n* (%)  Male  Female | 295 (48.5)  313 (51.5) | 210 (48.7)  221 (51.3) | 0.948 |
| Race, *n* (%)  Black  White | 308 (50.7)  300 (49.3) | 197 (45.7)  234 (54.3) | 0.116 |
| Socio-economic status, *n* (%)  Low  Medium  High | 232 (38.2)  167 (27.5)  209 (34.4) | 170 (39.5)  146 (34.0)  114 (26.5) | **0.013** |
| SES score, points | 21.03 (20.54; 21.52) | 20.30 (19.76; 20.84) | 0.050 |
| *Depression* |  |  |  |
| PHQ-9, total score | 7 (0, 27) | 8 (0, 23) | 0.099 |
| *Lifestyle-related descriptors* |  |  |  |
| *Total energy intake, kJ | 6464.81 (3351.76) | 6503.11 (3620.58) | 0.644 |
| *BMI, kg/m^2^ | 24.27 (6.66) | 24.03 (6.75) | 0.711 |

Data is presented as mean (95% CI), number of participants (%) or median (±IQR) for *non-parametric logarithmically transformed variables or mean (minimum to maximum range) for the depression score. Values in bold are regarded as statistically significant (*p* ≤ 0.05). Abbreviations: BMI, body mass index; PHQ, patient health questionnaire, SES, socioeconomic status.

**Supplementary Table 2. Average intake of dietary exposures at baseline of participants who completed the follow-up assessment compared with those who were enrolled at baseline but did not participate in the follow-up phase.**

| **Dietary exposures** | **Completed follow-up assessment**  ***n* = 608** | **Did not complete follow-up assessment**  ***n* = 431** | ***p*-value** |
| --- | --- | --- | --- |
| Fruit intake, g/day (*n* = 444) | 142.13 (147.39) | 150.21 (156.46) | 0.582 |
| Vegetable intake, g/day (*n* = 842) | 133.26 (106.20) | 129.82 (126.54) | 0.671 |
| Legumes intake, g/day (*n* = 102) | 55.17 (58.17) | 70.86 (114.40) | 0.409 |
| Wholegrain intake, g/day (*n* = 341) | 85.53 (91.06) | 85.90 (76.57) | 0.969 |
| Nuts and seeds intake, g/day (*n* = 61) | 31.73 (24.41) | 27.10 (17.58) | 0.527 |
| Milk intake, g/day (*n* = 719) | 164.70 (170.28) | 173.90 (140.39) | 0.445 |
| Red meat intake, g/day (*n* = 540) | 112.08 (90.19) | 119.46 (105.07) | 0.384 |
| Processed meat intake, g/day (*n* = 640) | 61.79 (56.58) | 71.84 (66.54) | **0.049** |
| Sugar-sweetened beverages intake (*n* = 641), g/day | 418.87 (352.88) | 453.33 (431.08) | 0.269 |
| Fibre intake, g/day (*n* = 1022) | 14.76 (7.75) | 14.83 (8.34) | 0.893 |
| Calcium intake, g/day (*n* = 1022) | 0.53 (0.38) | 0.50 (0.34) | 0.339 |
| Omega-3 fatty acid intake, g/day (*n* = 1022) | 184.79 (500.01) | 145.72 (316.36) | 0.157 |
| Polyunsaturated fat intake, % of TE (*n* = 1014) | 7.77 (3.65) | 7.84 (4.24) | 0.747 |
| Ultra-processed food intake, % of TE (*n* = 1014) | 45.69 (17.99) | 46.67 (18.36) | 0.399 |
| Non-ultra-processed food intake, % of TE (*n* = 1021) | 54.70 (18.19) | 53.78 (18.83) | 0.430 |

Data is presented as mean (SD). *n*-values represent the number of valid cases per dietary risk factor for the total cohort. Values in bold are regarded as statistically significant (*p* ≤ 0.05). Note: none of these results remained after adjustment for multiple comparisons.

**Supplementary Table 3. Sensitivity analyses of associations between dietary intake and depression after additional adjustment for BMI**

|  | ***n* = 306** | | |
| --- | --- | --- | --- |
| **Probability for depression** | **OR** | **95% CI** | ***p*-value** |
| Fruit per 100g | 0.905 | 0.740; 1.105 | 0.187 |
| Vegetables per 100g | 0.819 | 0.740; 1.000 | 0.062 |
| Wholegrains per 50g | 0.778 | 0.605; 0.951 | **0.014** |
| Milk per 10g | 1.010 | 1.000; 1.020 | 0.085 |
| Red meat per 25g | 0.975 | 0.905; 1.025 | 0.281 |
| Processed meat per 17g | 1.070 | 1.017; 1.126 | **0.007** |
| Sugar-sweetened beverages per 50g | 1.051 | 1.000; 1.051 | **0.011** |
| Fibre per 1g | 0.984 | 0.962; 1.005 | 0.137 |
| Calcium per 1g | 0.961 | 0.576; 1.605 | 0.880 |
| Omega-3 fatty acids per 1g | 1.000 | 1.000; 1.000 | 0.701 |
| PUFA per % of TE | 0.998 | 0.963; 1.034 | 0.925 |
| UPF per % of TE | 1.006 | 0.998; 1.014 | 0.148 |
| Non-UPF, per % of TE | 0.995 | 0.987; 1.002 | 0.182 |
|  |  | ***n* = 50** |  |
| **Relative risk for incident depression** | **RR** | **95% CI** | ***p*-value** |
| Fruit per 100g | 1.105 | 0.905; 1.349 | 0.305 |
| Vegetables per 1g | 1.221 | 0.905; 1.491 | 0.123 |
| Wholegrains per 50g | 0.905 | 0.740; 1.162 | 0.438 |
| Milk per 10g | 0.961 | 0.923; 0.990 | **0.018** |
| Red meat per 25g | 1.000 | 0.891; 1.133 | 0.915 |
| Processed meat per 17g | 1.070 | 0.983; 1.145 | 0.134 |
| Sugar-sweetened beverages per 50g | 0.951 | 0.905; 1.000 | **0.031** |
| Fibre per 1g | 1.001 | 0.960; 1.043 | 0.979 |
| Calcium per 1g | 0.869 | 0.267; 2.826 | 0.816 |
| Omega-3 fatty acids per 1mg | 1.000 | 1.000; 1.000 | 0.461 |
| PUFA per % of TE | 1.000 | 0.939; 1.066 | 0.996 |
| UPF per % of TE | 0.996 | 0.984; 1.008 | 0.510 |
| Non-UPF, per % of TE | 1.004 | 0.992; 1.017 | 0.497 |

Adjusted for body mass index in addition to sociodemographic information and total energy intake using Willetts residual method.

Abbreviations: CI, confidence interval; OR, odds ratio; PUFA, Polyunsaturated fat; UPF, Ultra-processed food intake. Values in bold are regarded as statistically significant (*p* ≤ 0.05).

Superscript * indicates results that remained after adjustment for multiple comparisons.

**Supplementary Table 4. Association between dietary intake and the relative risk of incident depression between baseline and follow-up**

| **Dietary exposures** | **Incident depression (*n* = 50)** | | |
| --- | --- | --- | --- |
| ***Model 1*** | **RR** | **95% CI** | ***p*-value** |
| Fruit per 100g | 1.000 | 0.819; 1.221 | 0.979 |
| Vegetables per 100g | 1.000 | 0.740; 1.349 | 0.967 |
| Wholegrains per 50g | 0.905 | 0.905; 1.162 | 0.355 |
| Milk per 10g | 0.951 | 0.914; 0.990 | **0.013** |
| Red meat per 25g | 0.951 | 0.839; 1.078 | 0.430 |
| Processed meat per 17g | 1.070 | 0.983; 1.165 | 0.163 |
| Sugar-sweetened beverages per 50g | 0.951 | 0.905; 1.000 | 0.200 |
| Fibre per 1g | 0.992 | 0.953; 1.032 | 0.689 |
| Calcium per 1g | 0.353 | 0.127; 0.976 | **0.045** |
| Omega-3 fatty acids per 1g | 1.000 | 1.000; 1.000 | 0.540 |
| PUFA per % of TE | 1.004 | 0.941; 1.071 | 0.912 |
| UPF per % of TE | 0.997 | 0.984; 1.009 | 0.601 |
| Non-UPF, per % of TE | 1.004 | 0.992; 1.017 | 0.495 |
| ***Model 2*** | **RR** | **95% CI** | ***p*-value** |
| Fruit per 100g | 1.105 | 0.816; 1.349 | 0.607 |
| Vegetables per 100g | 1.221 | 1.000; 1.491 | 0.118 |
| Wholegrains per 50g | 0.861 | 0.704; 1.105 | 0.23 |
| Milk per 10g | 0.961 | 0.923; 1.000 | 0.081 |
| Red meat per 25g | 1.000 | 0.882; 1.133 | 0.902 |
| Processed meat per 17g | 1.070 | 1.017; 1.145 | **0.019** |
| Sugar-sweetened beverages per 50g | 0.951 | 0.905; 1.000 | 0.189 |
| Fibre per 1g | 1.003 | 0.969; 1.038 | 0.879 |
| Calcium per 1g | 0.962 | 0.348; 2.661 | 0.941 |
| Omega-3 fatty acids per 1g | 1.000 | 1.000; 1.000 | 0.422 |
| PUFA per % of TE | 1.002 | 0.941; 1.066 | 0.957 |
| UPF per % of TE | 0.996 | 0.984; 1.008 | 0.503 |
| Non-UPF, per % of TE | 1.004 | 0.992; 1.017 | 0.489 |
| ***Model 3*** | **RR** | **95% CI** | ***p*-value** |
| Fruit per 100g | 1.000 | 0.819; 1.221 | 0.764 |
| Vegetables per 100g | 1.000 | 0.740; 1.349 | 0.961 |
| Wholegrains per 50g | 0.951 | 0.818; 1.105 | 0.549 |
| Milk per 10g | 0.942 | 0.914; 0.980 | **0.003*** |
| Red meat per 25g | 1.000 | 0.905; 1.105 | 0.935 |
| Processed meat per 17g | 1.052 | 0.950; 1.165 | 0.268 |
| Sugar-sweetened beverages per 50g | 0.951 | 0.905; 1.000 | 0.068 |
| Fibre per 1g | 0.996 | 0.959; 1.034 | 0.834 |
| Calcium per 1g | 0.327 | 0.114; 0.939 | **0.038** |
| Omega-3 fatty acids per 1g | 1.000 | 1.000; 1.000 | 0.512 |
| PUFA per % of TE | 1.004 | 0.942; 1.071 | 0.895 |
| UPF per % of TE | 0.997 | 0.984; 1.009 | 0.612 |
| Non-UPF, per % of TE | 1.004 | 0.991; 1.017 | 0.532 |
| ***Model 4*** | **RR** | **95% CI** | ***p*-value** |
| Fruit per 100g | 1.105 | 0.905; 1.349 | 0.410 |
| Vegetables per 1g | 1.221 | 0.905; 1.491 | 0.124 |
| Wholegrains per 50g | 0.905 | 0.778; 1.105 | 0.311 |
| Milk per 10g | 0.951 | 0.923; 0.990 | **0.015** |
| Red meat per 25g | 1.025 | 0.882; 1.161 | 0.822 |
| Processed meat per 17g | 1.070 | 0.983; 1.145 | 0.128 |
| Sugar-sweetened beverages per 50g | 0.951 | 0.905; 1.000 | **0.021** |
| Fibre per 1g | 0.998 | 0.959; 1.040 | 0.937 |
| Calcium per 1g | 0.820 | 0.254; 2.650 | 0.740 |
| Omega-3 fatty acids per 1mg | 1.000 | 1.000; 1.000 | 0.444 |
| PUFA per % of TE | 1.001 | 0.940; 1.066 | 0.970 |
| UPF per % of TE | 0.996 | 0.984; 1.008 | 0.489 |
| Non-UPF, per % of TE | 1.005 | 0.991; 1.017 | 0.460 |

Model 1 = unadjusted

Model 2 = adjusted for sociodemographic information (age, sex, ethnicity and socio-economic status).

Model 3 = adjusted for total energy intake using Willetts residual method.

Model 4 = adjusted for sociodemographic information and total energy intake using Willetts residual method.

Abbreviations: CI, confidence interval; RR, relative risk; PUFA, Polyunsaturated fat; UPF, Ultra-processed food intake. Values in bold are regarded as statistically significant (*p* ≤ 0.05).

Superscript * indicates results that remained after adjustment for multiple comparisons.
